# Correlation of changes in inflammatory and collagen biomarkers with durable guselkumab efficacy through 2 years in participants with active psoriatic arthritis: results from a phase III randomized controlled trial

**DOI:** 10.1177/1759720X241283536

**Published:** 2024-10-27

**Authors:** Stefan Siebert, Georg Schett, Siba P. Raychaudhuri, Monica Guma, Warner Chen, Sheng Gao, Soumya D. Chakravarty, Frederic Lavie, Proton Rahman

**Affiliations:** School of Infection & Immunity, University of Glasgow, Sir Graeme Davies Building, 4th Floor, 120 University Place, Glasgow G12 8TA, UK; Department of Medicine 3—Rheumatology and Immunology, Friedrich-Alexander-University Erlangen-Nürnberg and Universitätsklinikum Erlangen, Erlangen, Germany; Division of Rheumatology, Allergy and Clinical Immunology, University of California Davis, School of Medicine, Sacramento, CA, USA; VA Northern California Health Care System, Mather, CA, USA; Division of Rheumatology, Autoimmunity and Inflammation, Department of Medicine, University of California San Diego, La Jolla, CA, USA; Immunology, Janssen Research & Development, LLC, Spring House, PA, USA; Immunology, Janssen Research & Development, LLC, Spring House, PA, USA; Immunology, Janssen Scientific Affairs, LLC, a Johnson & Johnson Company, Horsham, PA, USA; Division of Rheumatology, Drexel University College of Medicine, Philadelphia, PA, USA; Immunology Global Medical Affairs, Janssen Cilag Global Medical Affairs, Issy les Moulineaux, France; Craig L. Dobbin Genetics Research Centre, Discipline of Medicine, Division of Rheumatology, Memorial University of Newfoundland, St John’s, Canada

**Keywords:** guselkumab, IL-23/Th-17 pathway, psoriatic arthritis, serum biomarkers

## Abstract

**Background:**

Guselkumab (human monoclonal antibody) selectively inhibits the interleukin (IL)-23p19 subunit.

**Objectives:**

Assess the longer-term pharmacodynamic effects of guselkumab and explore associations between such effects and clinical responses in patients with active psoriatic arthritis (PsA).

**Design:**

DISCOVER-2 randomized 739 biologic-naïve patients with active PsA (swollen/tender joint counts each ⩾5, C-reactive protein (CRP) ⩾0.6 mg/dL) to guselkumab (100 mg every 4 weeks (Q4W) or at Weeks 0, 4, and then Q8W) or placebo. Guselkumab-randomized participants with available serum biomarker data (randomly selected to reflect demographic and disease characteristics of the DISCOVER-2 population) comprised inflammatory (*N* = 100) and collagen (*N* = 178) biomarker cohorts.

**Methods:**

Pharmacodynamic effects of guselkumab through 2 years on inflammatory and collagen biomarker levels (general linear model) and associations between biomarkers and improvements in composite measures of joint, skin, and overall disease activity (Spearman linear regression) through 2 years were assessed. The relationship between the pharmacodynamic effects of guselkumab and achieving ⩾50% improvement in the American College of Rheumatology response criteria (ACR50) was assessed using a general linear model.

**Results:**

With guselkumab, pharmacodynamic effects on inflammatory (CRP, IL-6, serum amyloid A (SAA), IL-17A, IL-17F, IL-22, and beta-defensin 2 (BD-2)) and collagen (matrix metalloproteinase-degradation type I, III, IV, and VI collagen (C1M, C3M, C4M, and C6M)) biomarker levels were sustained or enhanced through Week 100. Throughout follow-up timepoints (Week 24/52/100), decreases in CRP, IL-6, C1M, and C6M levels correlated (*r* = 0.26–0.30; *p* < 0.05) with improved joint disease activity (Disease Activity in Psoriatic Arthritis); decreases in IL-17A, IL-17F, IL-22, and BD-2 levels correlated (*r* = 0.34–0.58; *p* < 0.05) with improved skin disease (Psoriasis Area and Severity Index); and decreases in C1M, C3M, C4M, and C6M correlated (*r* = 0.27–0.31; *p* < 0.05) with improved overall disease activity (Psoriatic Arthritis Disease Activity Score). Significantly (*p* < 0.05) greater reductions from baseline at Week 100 in CRP, IL-6, SAA, and C1M levels were observed in participants improving from Week 24 ACR50 nonresponse to Week 100 ACR50 response and were accompanied by a significant decrease in C1M from Week 24 to Week 100 versus nonresponders at both Weeks 24 and 100.

**Conclusion:**

In biologic-naïve participants with active PsA, guselkumab elicited substantial and enduring reductions in biomarkers that were associated with durable improvements in joint, skin, and overall disease activity through 2 years of DISCOVER-2.

**Trial registration:**

NCT03158285 (clinicaltrials.gov identifier).

## Introduction

Psoriatic arthritis (PsA) is a chronic, immune-mediated inflammatory disease that affects both skin and joints.^1^ Musculoskeletal involvement is common in PsA and can be characterized by joint tenderness and/or swelling, enthesitis, dactylitis, and bone loss^1^ that, if left untreated, can cause irreversible structural damage and substantial disability. Typically, the development of PsA is preceded by psoriasis and affects approximately 30% of these patients.^2^ Diagnosing PsA can be complicated by the heterogeneous nature of the disease, and symptoms may mimic those of other arthritic conditions such as osteoarthritis.^3^ In addition, as many as half of all patients with psoriasis may exhibit subclinical PsA signs and symptoms, such as joint pain and enthesitis. Thus, earlier recognition of the progression from psoriasis to PsA is important for determining effective treatment and improving overall outcomes.^4^ A better understanding of biomarkers in patients with PsA could be useful in diagnosing and monitoring affected patients.

Current treatment recommendations prioritize affected PsA disease domains of the individual patient, and common therapies include conventional synthetic disease-modifying antirheumatic drugs (csDMARDs), targeted synthetic DMARDs, and biologic DMARDs.^5^ Despite available drugs for the treatment of PsA, unmet needs exist for treatment strategies providing long-lasting efficacy across the PsA disease domains and for understanding the long-term pharmacodynamic effects of these therapies.

The pro-inflammatory cytokine interleukin (IL)-23 is a known key mediator of PsA pathogenesis.^6–8^ IL-23 acts upon T-helper (Th) 17 cells to induce the expression of effector cytokines including IL-17, IL-21, and IL-22.^9–11^ Available data indicate that IL-17 is a key factor in the development of psoriatic skin lesions,^12^ and IL-22, induced by IL-23 in the entheses, promotes enthesitis and pathologic bone formation.^13,14^ Furthermore, the production of IL-17A and tumor necrosis factor α (TNFα) within the IL-23/Th17 pathway may also lead to joint damage.^14^

Guselkumab (Janssen Biotech, Horsham, PA, USA) is a fully human monoclonal IgG1λ antibody that selectively inhibits the IL-23p19 subunit. Guselkumab 100 mg every 4 weeks (Q4W) or every 8 weeks (Q8W) demonstrated significantly greater efficacy versus placebo across disease domains among participants with active PsA in the phase III DISCOVER-1^15^ and DISCOVER-2^16^ trials, with durable responses seen through 1^17,18^ and 2^19^ years of treatment. Furthermore, at Week 24 of DISCOVER-2, inhibition of radiographic progression was statistically significant in participants treated with guselkumab 100 mg Q4W,^16^ and low rates of radiographic progression were sustained through 1^18^ and 219 years with both guselkumab dosing regimens.

The efficacy of guselkumab in treating the signs and symptoms of PsA was supported by observed pharmacodynamic effects in the DISCOVER-1 and DISCOVER-2 studies. Baseline levels of acute phase proteins (C-reactive protein (CRP), serum amyloid A (SAA), IL-6), and IL-23/Th17 effector cytokines (IL-17A, IL-17F, IL-22) in the pooled DISCOVER-1 and DISCOVER-2 cohorts as well as biomarkers of collagen degradation in the DISCOVER-2 cohort were elevated in patients with active PsA in comparison with healthy controls.^20,21^ Treatment with both guselkumab 100 mg Q4W and Q8W resulted in significant decreases from baseline, as early as Week 4, in levels of IL-17A, IL-17F, and IL-22 (pooled DISCOVER-1 and DISCOVER-2)^20^ and in collagen biomarkers (DISCOVER-2).^21^ Further decreases from baseline in these inflammatory^20^ and collagen^21^ biomarker levels were seen with guselkumab at Week 24, and decreases in collagen biomarker levels were maintained at Week 52.^21^ To date, no studies have evaluated biomarker levels beyond 1 year in patients with PsA receiving an IL-23 inhibitor.

To further characterize the longer-term pharmacodynamic effects of guselkumab in biologic-naïve patients with active PsA and to understand the molecular mechanism behind the observed durable clinical efficacy with guselkumab, these post hoc analyses of DISCOVER-2 assessed serum levels of inflammatory and collagen turnover biomarkers through 2 years of guselkumab treatment. Relationships between the extended pharmacodynamic effects of guselkumab and long-term clinical responses were also explored.

## Methods

### Study design and population

The study design and participant eligibility criteria for DISCOVER-2 (clinicaltrials.gov Identifier: NCT03158285) were previously described.^16^ Briefly, DISCOVER-2 was a phase III, randomized, placebo-controlled study that was conducted in 118 sites in 13 countries (Asia, Europe, and North America). The study enrolled adults who met the ClASsification for Psoriatic ARthritis (CASPAR) criteria and had active disease (swollen joint count (SJC) ⩾5, tender joint count (TJC) ⩾5, and CRP ⩾0.6 mg/dL) despite treatment with standard nonbiologic therapies. Participants also had current (⩾1 psoriatic plaque ⩾2 cm in diameter or nail changes consistent with psoriasis) or a documented history of psoriasis. Exclusion criteria included a diagnosis for another inflammatory disease that might confound the evaluations and prior exposure to biologics and Janus kinase inhibitors. Participants were randomly assigned (1:1:1) to receive subcutaneous injections of guselkumab 100 mg Q4W; guselkumab 100 mg at Week 0, Week 4, and then Q8W; or placebo with crossover to guselkumab 100 mg Q4W at Week 24. The final study drug administration was at Week 100.

**Figure 1.**
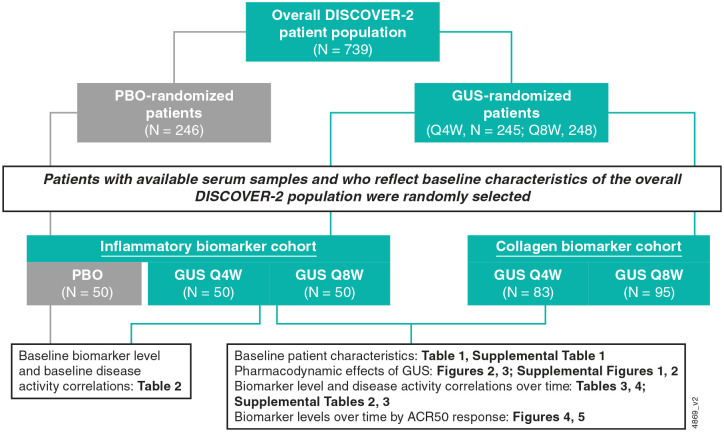
Disposition of biologic-naïve adults with active PsA from DISCOVER-2 included in post hoc biomarker analyses. Patients with available serum samples at Weeks 0 (baseline), 4, 24, 52, and 100 and who reflect baseline characteristics of the overall DISCOVER-2 patient population were randomly selected for the inflammatory and collagen biomarker cohorts. ACR50, ⩾50% improvement in American College of Rheumatology response criteria; GUS, guselkumab; PBO, placebo, PsA, psoriatic arthritis; Q4W, every 4 weeks; Q8W, every 8 weeks.

These post hoc analyses were conducted utilizing data from DISCOVER-2 participants who were previously included in one or both of the independently established and non-mutually exclusive biomarker cohorts.^20,21^ Serum samples were to be collected from all DISCOVER-2 participants per protocol. Among those with available serum samples at Weeks 0, 4, 24, 52, and 100, a total of 150 (50 per treatment arm) were randomly selected, while maintaining consistency with baseline characteristics of the overall DISCOVER-2 population, for inclusion in analyses of serum inflammatory biomarkers (inflammatory biomarker cohort; Figure 1).^20^ Similarly, among participants with available serum samples at Weeks 0, 4, 24, 52, and 100, a total of 178 were randomly selected from the guselkumab Q4W (*N* = 83) and Q8W (*N* = 95) groups for analyses of collagen turnover protein concentrations (collagen biomarker cohort; Figure 1). A larger sample size was selected for the collagen biomarker cohort to decrease variability.^21^

DISCOVER-2 was conducted in accordance with the Declaration of Helsinki and Good Clinical Practice guidelines, and all patients gave written informed consent.

### Biomarker assessments

Serum samples were collected as previously described.^20^ Briefly, serum was separated from blood samples in serum separation tubes, aliquoted, and frozen at −20°C or lower. Samples were shipped on dry ice to Covance Central Laboratory Services (Indianapolis, IN, USA) for storage prior to biomarker measurement and then shipped to Janssen Research & Development Biobank (Spring House, PA, USA).

Serum concentrations of biomarkers were quantified among DISCOVER-2 participants with evaluable samples. Inflammatory biomarkers included the acute phase proteins CRP, IL-6, SAA, and TNFα (Meso Scale Discovery (MSD) Platform, Rockville, MD, USA); IL-23/Th17 effector cytokines IL-17A, IL-17F, and IL-22 (Single Molecule Counting Erenna Immunoassay Platform, Millipore, Burlington, MA, USA); and the antimicrobial peptide β-defensin 2 (BD-2; MSD Platform), a target of the IL-23/Th17 pathway that is abundant in keratinocytes.^22^ In a prior post hoc analysis of biomarker data from DISCOVER-2, levels of matrix metalloproteinase (MMP)-degradation types I, III, IV, and VI collagen (C1M, C3M, C4M, and C6M, respectively) were modulated by guselkumab over time.^21^ Thus, these collagen biomarkers were assessed in the present analyses, and serum concentrations were quantified by Nordic Bioscience (Herlev, Denmark) as previously described.^21^

### Clinical efficacy assessments and outcome measures

The following clinical efficacy assessments were performed and patient-reported outcomes were collected as previously described^16^: SJC (66 joints), TJC (68 joints), Dactylitis Severity Score (DSS; 20 digits, each scored from 0 (no dactylitis) to 3 (severe dactylitis); total score 0–60),^23^ Leeds Enthesitis Index (LEI; 0–6),^24^ Physician Global Assessment (PhGA; 0–10 visual analog scale (VAS)),^25^ Psoriasis Area and Severity Index (PASI; 0–72),^26^ patient-reported pain (Patient Pain; 0–10 VAS), Patient Global Assessment of arthritis (PtGA Arthritis; 0–10 VAS), PtGA of arthritis and psoriasis (PtGA Arthritis + Psoriasis; 0–100 VAS), Health Assessment Questionnaire-Disability Index (HAQ-DI; 0–3),^27^ and the 36-item Short-Form Health Survey Physical Component Summary (SF-36 PCS) score (lower score indicates worse health-related quality of life).^28^

Several composite outcome measures were utilized in the current analyses. The Disease Activity in PSoriatic Arthritis (DAPSA) score (composite of SJC and TJC, PtGA Arthritis, Patient Pain, and serum CRP level)^29^ and clinical DAPSA (cDAPSA) score (excludes serum CRP level)^30^ primarily assess joint disease activity. Achievement of ⩾50% improvement in American College of Rheumatology response criteria (ACR50; ⩾50% improvement from baseline in both SJC and TJC, and ⩾50% improvement in ⩾3 of the following: serum CRP level, Patient Pain, PtGA Arthritis, PhGA, and HAQ-DI) is a stringent joint efficacy response that incorporates laboratory and physical function assessments.^25^ The PsA Disease Activity Score (PASDAS), a composite measure comprehensive of the key PsA domains, combines weighted assessments of SJC and TJC, serum CRP level, PtGA Arthritis + Psoriasis (0–100 VAS), PhGA (0–100 VAS), LEI, dactylitis digit count (0–20), and SF-36 PCS score.^31^

### Data analyses

Baseline demographic and disease characteristics were described for participants in the guselkumab Q4W, guselkumab Q8W, and combined guselkumab (Q4W + Q8W) groups of the overall DISCOVER-2 study population and for the inflammatory and collagen biomarker cohorts.

#### Correlations between baseline biomarker levels and PsA disease activity

Combining data from participants across the three randomized treatment groups in the inflammatory biomarker cohort, correlations between log_2_-transformed (for normalization of data distribution) baseline inflammatory biomarker levels (i.e., acute phase proteins, IL-23/Th17 effector cytokines, and BD-2) and baseline measures of disease activity (i.e., DAPSA, cDAPSA, PASI, PASDAS) were assessed using Spearman linear regression. Participants with missing data at baseline for a specific biomarker were excluded from these analyses. Statistically significant correlations were defined as having an absolute *rho* value exceeding 0.25 (*r* > 0.25) and *p* < 0.05.

#### Pharmacodynamic effect of guselkumab on biomarker levels over time

Changes in log_2_-transformed inflammatory biomarker levels from baseline at Weeks 4, 24, 52, and 100 with combined guselkumab Q4W + Q8W and with the individual guselkumab Q4W and Q8W dosing regimens were determined using a general linear model in both the inflammatory and collagen biomarker cohorts. No imputation was performed for missing biomarker concentrations.

#### Associations between changes in biomarker levels and disease activity with guselkumab

Changes from baseline at Weeks 24, 52, and 100 in DAPSA, cDAPSA, PASI, and PASDAS, and the above-mentioned components of the composite measures were determined for guselkumab-randomized (combined Q4W + Q8W and individual Q4W and Q8W dosing regimens) participants in the inflammatory and collagen biomarker cohorts employing observed data for both clinical measures and biomarker levels.

DISCOVER-2 was powered to assess efficacy by the achievement of an ACR20 response at Week 24 as previously described^16^; all findings from biomarker analyses are considered exploratory. Spearman linear regression was used to assess correlations between changes in inflammatory and collagen biomarkers and changes in PsA disease activity (DAPSA, cDAPSA, PASI, PASDAS, and components) at the individual Week 24, 52, and 100 timepoints and across all timepoints pooled. Spearman linear regression was also used to assess correlations between changes in inflammatory biomarker levels (i.e., acute phase proteins, IL-23/Th17 effector cytokines, and BD-2) at Week 24 and changes in disease activity (DAPSA, cDAPSA, PASI, and PASDAS) at Week 100. Statistically significant correlations were defined by *r* > 0.25 and *p* < 0.05.

#### Biomarker levels by ACR50 response to guselkumab

To assess the impact of earlier pharmacodynamic effects on subsequent achievement of stringent control of arthritis disease activity, levels of inflammatory and collagen biomarkers through Week 24 were compared for Week 100 ACR50 responders and nonresponders in the respective biomarker cohort using a general linear model, assuming normal distributions for each group. The prior analyses of ACR50 response rates in DISCOVER-2 utilized nonresponder imputation for participants meeting treatment failure rules through Week 24^16^ or with missing data through Week 100^19^; however, no imputation was performed for missing biomarker data. Therefore only those participants with observed values for both ACR50 response and biomarker levels were included in this analysis.

To assess the relationship between pharmacodynamic effects and control of joint disease activity, mean changes in the aforementioned inflammatory and collagen biomarker levels from baseline at Weeks 4, 24, 52, and 100 and from Week 24 to Weeks 52 and 100 were assessed by ACR50 response achievement using a general linear model. Participants were classified by achievement of ACR50 response (R) or nonresponse (NR) into one of the following cohorts: those who achieved ACR50 response at both Weeks 24 and 100 (R/R), those who did not achieve an ACR50 response at Week 24 but did so at Week 100 (NR/R), or those who did not achieve an ACR50 response at either Week 24 or 100 (NR/NR). Participants who achieved ACR50 response at Week 24 but not at Week 100 (R/NR) were not included in this analysis due to the small number of participants meeting these criteria. Statistically significant changes in biomarker levels from baseline at Weeks 24, 52, and 100 and from Week 24 at Weeks 52 and 100 within each ACR50 response cohort were defined as those having *p* < 0.05 and absolute fold difference ⩾1.4 for inflammatory biomarkers or ⩾1.25 for collagen biomarkers. For participants who did not achieve an ACR50 response at Week 24, changes in levels of inflammatory and collagen biomarkers were compared between those who later achieved this response at Week 100 (NR/R) and those who did not (NR/NR), using the same criteria for statistical significance. Changes in biomarker levels were not formally tested for the other ACR50 responder cohorts.

## Results

### Patient cohorts and baseline characteristics

In DISCOVER-2, 739 participants were randomized and received study treatment: 493 in the combined guselkumab Q4W + Q8W-randomized group and 246 in the placebo group. For these post hoc analyses, 100 guselkumab-randomized (50 from each guselkumab treatment group), along with 50 placebo-randomized participants (included for baseline analyses), comprised the inflammatory biomarker cohort. The collagen biomarker group included 178 guselkumab-randomized participants (Q4W 83; Q8W 95; Figure 1).

Consistent with the process for cohort patient selection, baseline demographic and disease characteristics of guselkumab-randomized participants (combined Q4W + Q8W) were generally similar between the inflammatory and collagen biomarker cohorts (Table 1). Respective baseline mean DAPSA scores (50.0 and 50.3), PASI scores (10.2 and 10.1), and PASDAS (6.7 and 6.7) within the inflammatory and collagen biomarker cohorts indicated highly active joint disease, consistent with DISCOVER-2 inclusion criteria, and moderately active psoriasis and overall PsA disease activity. The frequency of baseline concomitant medication use in the inflammatory and collagen biomarker cohorts was also comparable with that of the overall DISCOVER-2 population for csDMARDs (67.0%–69.7%) and corticosteroids (18.5%–22.0%). Baseline characteristics were largely similar between the individual guselkumab Q4W and Q8W groups within the inflammatory and biomarker cohorts (Supplemental Table 1).

**Table 1.** Baseline demographic and disease characteristics: guselkumab (Q4W + Q8W)-randomized participants in the overall, inflammatory, and collagen biomarker cohorts of DISCOVER-2.

Baseline characteristic	Overall DISCOVER-2	Inflammatory biomarker cohort	Collagen biomarker cohort
Participants, *n*	493	100	178
Age, years	45.4 (11.7)	46.3 (11.6)	45.1 (11.2)
Male, %	55.0	54.0	57.9
White, %	97.8	98.0	96.6
BMI, kg/m^2^	28.9 (6.1)	28.6 (5.6)	29.0 (5.9)
CRP, mg/dL			
Median (IQR)	1.2 (0.0–19.0)	1.6 (0.0–10.3)	1.5 (0.1–19.0)
Mean (SD)	1.9 (2.3)	2.1 (2.2)	2.1 (2.3)
SJC (0–66)	12.3 (7.4)	13.0 (7.9)	13.1 (8.4)
TJC (0–68)	21.1 (12.8)	22.0 (12.9)	22.2 (13.5)
DSS (1–60)	8.3 (9.6)^a^	9.3 (10.1)^a^	9.2 (11.6)^a^
LEI (1–6)	2.8 (1.6)^b^	3.0 (1.7)^b^	2.7 (1.5)^b^
PhGA (VAS; 0–10)	6.6 (1.6)	6.7 (1.5)	6.8 (1.5)
PtGA arthritis (VAS; 0–10)	6.5 (1.9)	6.5 (2.0)	6.5 (1.8)
PtGA arthritis + PsO (VAS; 0–100)	67.3 (20.0)	69.0 (19.4)	67.5 (19.2)
Patient pain (VAS; 0–10)	6.2 (2.0)	6.4 (2.0)	6.3 (1.8)
HAQ-DI (0–3)	1.3 (0.6)	1.3 (0.6)	1.3 (0.6)
DAPSA score	48.0 (20.3)	50.0 (21.2)	50.3 (21.8)
cDAPSA score	46.1 (19.9)	47.9 (20.7)	48.2 (21.2)
PASI score (0–72)	10.2 (11.7)	10.2 (11.9)	10.1 (11.2)
PASDAS (0–10)	6.6 (1.1)	6.7 (1.1)	6.7 (1.1)
Concomitant medications, %			
csDMARD	69.0	67.0	69.7
Methotrexate	58.2	57.0	60.1
Corticosteroid	19.5	22.0	18.5

Data are mean (SD) unless otherwise indicated.

a Among participants with dactylitis at baseline (overall DISCOVER-2, *N* = 232; inflammatory biomarker cohort, *N* = 44; collagen biomarker cohort, *N* = 89).

b Among participants with enthesitis at baseline (overall DISCOVER-2, *N* = 323; inflammatory biomarker cohort, *N* = 66; collagen biomarker cohort, *N* = 123).

BMI, body mass index; cDAPSA, clinical DAPSA; CRP, C-reactive protein; csDMARD, conventional synthetic disease-modifying antirheumatic drug; DAPSA, Disease Activity in Psoriatic Arthritis; DSS, dactylitis severity score; HAQ-DI, Health Assessment Questionnaire-Disability Index; IQR, interquartile range; LEI, Leeds enthesitis index; PASDAS, Psoriatic Arthritis Disease Activity Score; PASI, Psoriasis Area and Severity Index; PhGA, Physician Global Assessment; PsO, psoriasis; PtGA, Patient Global Assessment; Q4W, every 4 weeks; Q8W, every 8 weeks; SD, standard deviation; SJC, swollen joint count; TJC, tender joint count; VAS, visual analog scale.

### Correlations between biomarker levels and disease activity at baseline

In the full inflammatory biomarker cohort, correlations at baseline (prior to any study agent administration) were observed between DAPSA score and CRP and IL-6; cDAPSA score and IL-6; PASI score and IL-17A, IL-17F, IL-22, and BD-2; and between PASDAS and CRP, SAA, and IL-6 concentrations (Table 2).

**Table 2.** Correlations between serum biomarker levels and disease activity at baseline: all randomized participants in the inflammatory biomarker cohort of DISCOVER-2.

Serum cytokine	DAPSA	cDAPSA	PASI	PASDAS
CRP	**0.28**	0.19	0.05	**0.30**
IL-6	**0.36**	**0.31**	0.04	**0.29**
SAA	0.18	0.11	0.02	**0.27**
TNFα	0.15	0.14	0.23	0.14
IL-17A	0.02	0.009	**0.40**	0.08
IL-17F	−0.01	−0.02	**0.43**	0.08
IL-22	0.06	0.04	**0.37**	0.20
BD-2	0.00	−0.004	**0.64**	0.14

Among participants with available biomarker data (guselkumab Q4W, *N* = 50; guselkumab Q8W, *N* = 50; placebo, *N* = 50). Bolded rho (*r*) values represent a significant correlation between cytokine levels and disease activity score (*r* > 0.25 and *p* < 0.05).

BD-2, β-defensin 2; cDAPSA, clinical DAPSA; CRP, C-reactive protein; DAPSA, Disease Activity in Psoriatic Arthritis; IL, interleukin; PASDAS, Psoriatic Arthritis Disease Activity Score; PASI, Psoriasis Area and Severity Index; Q4W, every 4 weeks; Q8W, every 8 weeks; SAA, serum amyloid A; TNFα, tumor necrosis factor α.

### Pharmacodynamic effects of guselkumab through Week 100

**Figure 2.**
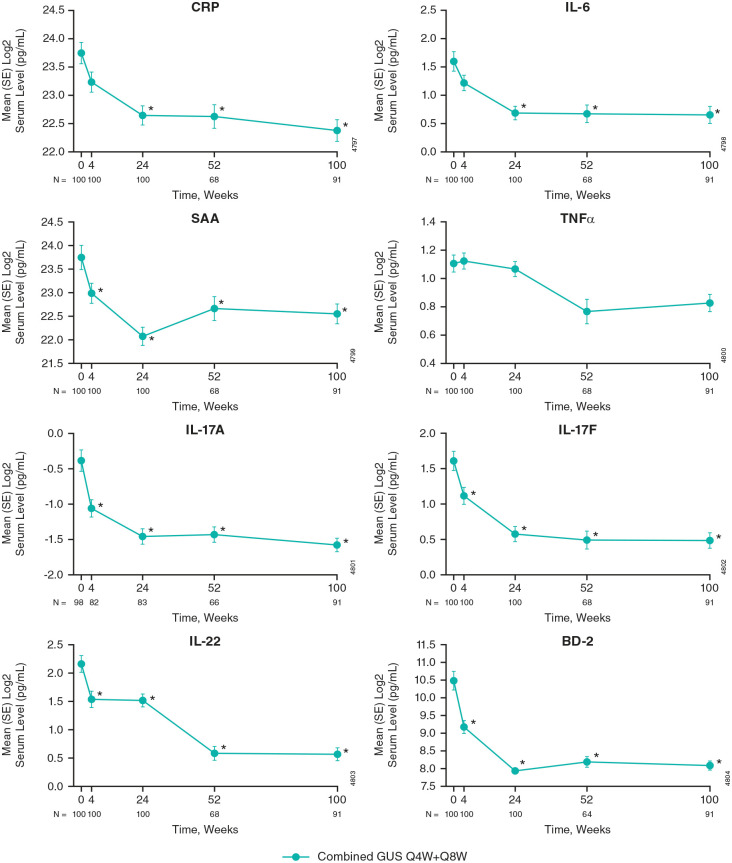
Serum levels of inflammatory biomarkers from baseline through Week 100 with guselkumab treatment. Among DISCOVER-2 participants randomized to guselkumab 100 mg (combined Q4W+Q8W) in the inflammatory biomarker cohort. Statistics are based on a general linear model. *Indicates statistically significant change from baseline (*p* < 0.05 and **|**fold difference**|** ⩾1.4). BD-2, β-defensin 2; CRP, C-reactive protein; GUS, guselkumab; IL, interleukin; Q4W, every 4 weeks; Q8W, every 8 weeks; SAA, serum amyloid A; SE, standard error; TNFα, tumor necrosis factor α.

As early as Week 4, concentrations of SAA, IL-17A, IL-17F, IL-22, and BD-2 were statistically significantly lower than those at baseline (Figure 2). These levels further decreased at Week 24, when CRP and IL-6 levels were also statistically significantly lower than at baseline. Reductions in inflammatory biomarkers through Week 24 were generally sustained through Week 100. Although TNFα levels generally remained stable through Week 24, reductions from baseline observed between Weeks 24 and 52 were sustained through Week 100 (Figure 2).

**Figure 3.**
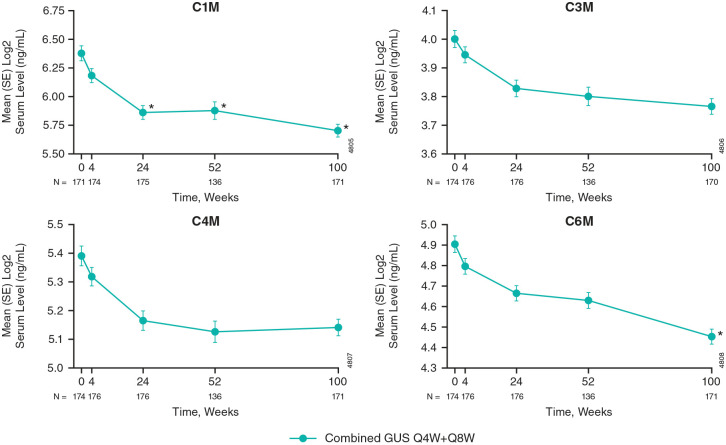
Serum levels of collagen biomarkers from baseline through Week 100 with guselkumab treatment. Among DISCOVER-2 participants randomized to guselkumab 100 mg (combined Q4W + Q8W) in the collagen biomarker cohort. Statistics are based on a general linear model. *Indicates statistically significant change from baseline (*p* < 0.05 and |fold difference| ⩾1.25). C1M, MMP-degradation type 1 collagen; C3M, MMP-degradation type III collagen; C4M, MMP-degradation type IV collagen; C6M, MMP-degradation type VI collagen; GUS, guselkumab; Q4W, every 4 weeks; Q8W, every 8 weeks; MMP, matrix metalloproteinase; SE, standard error.

Of the collagen biomarkers assessed, statistically significant reductions from baseline were observed for C1M (Week 24) and C6M (Week 100) levels (Figure 3). Decreases from baseline in C3M and C4M through Week 100 did not reach statistical significance. These trends were similar in the individual guselkumab Q4W and Q8W treatment groups to those observed in the combined guselkumab Q4W + Q8W group (Supplemental Figures 1 and 2).

### Correlations between changes in biomarker levels and disease activity over time among guselkumab-randomized participants

For joint disease activity scores, reductions from baseline in serum CRP and SAA levels at Week 100 of guselkumab treatment positively correlated with improvements in DAPSA and cDAPSA scores (Table 3). Decreases in C6M levels at Weeks 24 and 100 and in C1M and C4M levels at Week 52 also correlated with decreases in DAPSA score at these time points. Reductions from baseline in serum IL-6 levels at Week 24 correlated with later Week 100 improvements from baseline in DAPSA and cDAPSA scores (Table 4).

**Table 3.** Correlations between changes from baseline in inflammatory and collagen biomarker levels and changes from baseline in disease activity measures across three timepoints (Weeks 24, 52, and 100) and pooled timepoints: guselkumab (Q4W+Q8W)-randomized participants from the inflammatory and collagen biomarker cohorts of DISCOVER-2.

Time	Biomarker	DAPSA	cDAPSA	PASI	PASDAS
Week 24	CRP	**0.27**	0.22	−0.03	0.20
	IL-6	**0.25**	0.20	−0.03	0.19
	SAA	0.16	011	0.01	0.19
	TNFα	0.05	0.04	0.14	0.09
	IL-17A	0.04	0.04	**0.44**	**0.28**
	IL-17F	0.02	0.03	**0.41**	0.15
	IL-22	0.14	0.15	**0.41**	0.15
	BD-2	0.12	0.14	**0.61**	**0.26**
	C1M	**0.25**	0.18	0.06	0.19
	C3M	0.22	0.15	0.09	0.23
	C4M	0.22	0.15	0.10	0.22
	C6M	**0.29**	0.23	0.07	**0.28**
Week 52	CRP	**0.33**	**0.26**	0.09	0.18
	IL-6	**0.33**	**0.27**	−0.03	0.11
	SAA	0.19	0.14	0.24	0.15
	TNFα	0.08	0.01	−0.04	0.03
	IL-17A	0.00	−0.04	**0.43**	0.08
	IL-17F	−0.09	−0.11	**0.41**	−0.02
	IL-22	−0.05	−0.09	**0.26**	−0.10
	BD-2	0.10	0.09	**0.55**	0.15
	C1M	**0.26**	0.20	0.07	**0.28**
	C3M	0.19	0.13	−0.03	0.24
	C4M	**0.26**	0.19	0.01	**0.33**
	C6M	0.21	0.14	0.05	**0.26**
Week 100	CRP	**0.32**	**0.28**	0.19	**0.33**
	IL-6	0.22	0.17	−0.01	0.22
	SAA	**0.32**	**0.28**	0.21	**0.33**
	TNFα	0.15	0.13	0.21	0.24
	IL-17A	0.13	0.13	**0.51**	0.21
	IL-17F	−0.01	0.004	**0.39**	0.11
	IL-22	0.06	0.06	**0.31**	0.13
	BD-2	0.04	0.04	**0.59**	0.11
	C1M	0.22	0.17	0.07	**0.29**
	C3M	0.22	0.16	0.08	**0.29**
	C4M	0.16	0.10	0.07	**0.25**
	C6M	**0.27**	0.22	−0.01	**0.29**
Pooled Weeks 24, 52, and 100	CRP	**0.30**	0.24	0.08	0.24
	IL-6	**0.26**	0.21	−0.03	0.17
	SAA	0.20	0.14	0.14	0.20
	TNFα	0.16	0.13	0.14	0.19
	IL-17A	0.08	0.07	**0.46**	0.21
	IL-17F	0.00	−0.01	**0.41**	0.11
	IL-22	0.17	0.17	**0.34**	0.22
	BD-2	0.08	0.08	**0.58**	0.16
	C1M	**0.27**	0.21	0.07	**0.27**
	C3M	0.24	0.18	0.06	**0.27**
	C4M	0.23	0.16	0.06	**0.27**
	C6M	**0.30**	0.24	0.04	**0.31**

Among participants with available biomarker data (inflammatory biomarker cohort, *N* = 100; collagen biomarker cohort, *N* = 178). Bolded rho (*r*) values represent a statistically significant correlation between cytokine levels and disease activity score (*r* > 0.25 and *p* < 0.05).

BD-2, β-defensin 2; C1M, MMP-degradation type 1 collagen; C3M, MMP-degradation type III collagen; C4M, MMP-degradation type IV collagen; C6M, MMP-degradation type VI collagen; cDAPSA, clinical DAPSA; CRP, C-reactive protein; DAPSA, Disease Activity in Psoriatic Arthritis; IL, interleukin; PASDAS, Psoriatic Arthritis Disease Activity Score; PASI, Psoriasis Area and Severity Index; MMP, matrix metalloproteinase; Q4W, every 4 weeks; Q8W, every 8 weeks; SAA, serum amyloid A; TNFα, tumor necrosis factor α.

**Table 4.** Correlations between changes from baseline at Week 24 in inflammatory biomarker levels and changes from baseline at Week 100 in disease activity: guselkumab (Q4W+Q8W)-randomized participants from the inflammatory biomarker cohort of DISCOVER-2.

Inflammatory biomarker	DAPSA	cDAPSA	PASI	PASDAS
Acute phase proteins				
CRP	0.22	0.18	−0.05	0.22
IL-6	**0.32**	**0.28**	−0.03	0.19
SAA	0.17	0.13	0.06	0.21
IL-23/Th17 effector cytokines				
IL-17A	0.03	0.04	**0.41**	0.06
IL-17F	0.02	0.01	**0.41**	0.07
IL-22	−0.03	−0.03	**0.38**	0.04
Antimicrobial peptide				
BD-2	0.10	0.11	**0.60**	0.13

Among participants with available biomarker data (*N* = 100; *n* = 50 for each guselkumab regimen). Bolded rho (*r*) values represent a statistically significant correlation between cytokine levels and disease activity score (*r* > 0.25 and *p* < 0.05).

BD-2, β-defensin 2; cDAPSA, clinical DAPSA; CRP, C-reactive protein; DAPSA, Disease Activity in Psoriatic Arthritis; IL, interleukin; PASDAS, Psoriatic Arthritis Disease Activity Score; PASI, Psoriasis Area and Severity Index; Q4W, every 4 weeks; Q8W, every 8 weeks; SAA, serum amyloid A.

For skin psoriasis activity, decreases in 17A, IL-17F, IL-22, and BD-2 levels with guselkumab, at all time points assessed, correlated with concurrent improvements in PASI score (Table 3). Changes from baseline in these same biomarkers at Week 24 correlated with future changes from baseline in PASI score at Week 100 (Table 4).

For overall PsA disease activity, correlations were seen between changes in C6M (Weeks 24, 52, and 100), in C1M and C4M (Weeks 52 and 100), and in CRP and SAA (Week 100) levels and changes in PASDAS at these same timepoints (Table 3). Week 24 biomarker changes did not predict later changes in PASDAS at Week 100 (Table 4).

When pooling data across Weeks 24, 52, and 100, reductions in levels of acute phase proteins (CRP, IL-6) and collagen biomarkers (C1M, C6M) with guselkumab treatment correlated with improvements in DAPSA; reductions in IL-23/Th17 effector cytokines (IL-17A, IL-17F, IL-22) and BD-2 correlated with improvements in PASI; and reductions in C1M, C3M, C4M, and C6M levels correlated with improvements in PASDAS (Table 3). Relationships observed between inflammatory and collagen biomarker levels and improvements in joint, skin, and overall PsA disease activity in the combined guselkumab Q4W + Q8W group were similar in the individual guselkumab Q4W and Q8W groups; although some associations did not reach statistical significance (Supplemental Table 2).

Changes in biomarker levels with guselkumab also correlated with changes in components of the composite indices at individual and pooled timepoints. Specifically, changes in acute phase proteins correlated with changes in CRP levels, TJC, PtGA (Arthritis and Arthtitis + Psoriasis), and SF-36 PCS at Week 100, and changes in collagen degradation biomarkers consistently correlated with changes in CRP (C1M, C3M, C4M, C6M), Patient Pain (C6M), and SF-36 PCS score (C6M; Supplemental Table 3).

### Biomarker levels by ACR50 response to guselkumab

**Figure 4.**
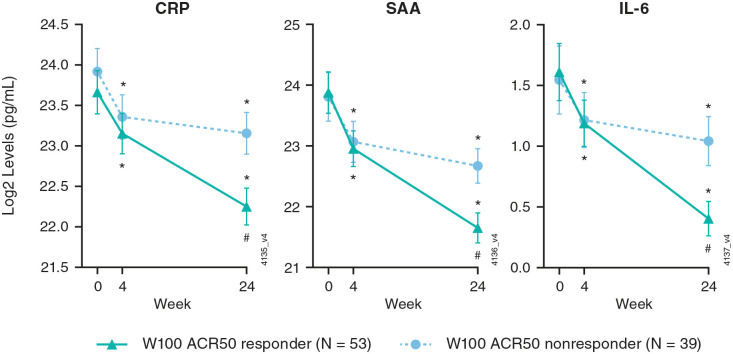
Serum levels of acute phase proteins through Week 24 for Week 100 ACR50 responders and nonresponders. Data from DISCOVER-2 participants randomized to guselkumab 100 mg (combined Q4W + Q8W) in the inflammatory biomarker cohort. Statistics are based on a general linear model. *Indicates statistically significant change from baseline (*p* < 0.05). ^#^Indicates statistically significant difference compared to W100 ACR50 NR (*p* < 0.05). ACR50, ⩾50% improvement in American College of Rheumatology response criteria; CRP, C-reactive protein; IL, interleukin; NR, nonresponder; Q4W, every 4 weeks; Q8W, every 8 weeks; SAA, serum amyloid A; W, week.

At Week 100, 58% and 54% of guselkumab-randomized (combined Q4W + Q8W) participants of the inflammatory and collagen biomarker cohorts, respectively, achieved an ACR50 response. Relationships between biomarker levels and long-term (Week 100) achievement of ACR50 with guselkumab were assessed in the inflammatory and collagen biomarker cohorts. In both ACR50 responders and nonresponders at Week 100, serum concentrations of CRP, SAA, and IL-6 at Weeks 4 and 24 were statistically significantly lower than those at baseline; however, levels at Week 24 were significantly lower among responders than in nonresponders (Figure 4).

**Figure 5.**
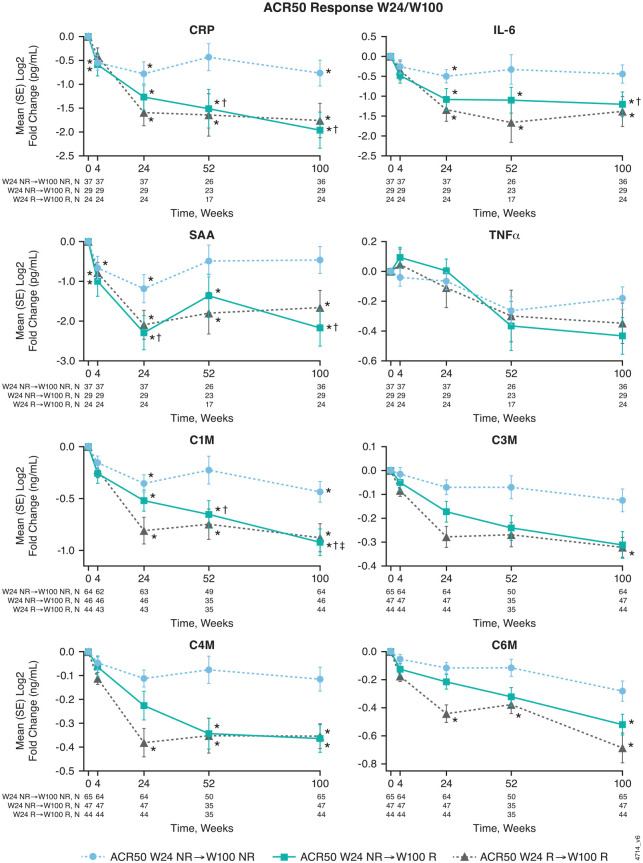
Changes in serum levels of select inflammatory and collagen biomarkers from baseline through Week 100 with guselkumab treatment by patterns of Week 24 and 100 ACR50 response. Among DISCOVER-2 participants randomized to guselkumab 100 mg (combined Q4W + Q8W) in the inflammatory biomarker (for CRP, IL-6, SAA, TNFα) and collagen biomarker (for C1M, C3M, C4M, C6M) cohorts. Statistics are based on a general linear model. *Indicates statistically significant change from baseline (*p* < 0.05 and **|** fold difference **|** ⩾1.4 (inflammatory biomarkers) or ⩾1.25 (collagen biomarkers)). ^†^Indicates statistically significant difference between ACR50 W24 NR→W100 R compared with ACR50 W24 NR→W100 NR (*p* < 0.05 and **|** fold difference **|** ⩾1.4 (inflammatory biomarkers) or ⩾1.25 (collagen biomarkers)). ^‡^Indicates statistically significant change from W24 (*p* < 0.05 and | fold difference | ⩾1.25). ACR50, ⩾50% improvement in American College of Rheumatology response criteria; C1M, MMP-degradation type 1 collagen; C3M, MMP-degradation type III collagen; C4M, MMP-degradation type IV collagen; C6M, MMP-degradation type VI collagen; CRP, C-reactive protein; IL, interleukin; MMP, matrix metalloproteinase; NR, nonresponder; Q4W, every 4 weeks; Q8W, every 8 weeks; R, responder; SAA, serum amyloid A; SE, standard error; TNFα, tumor necrosis factor α; W, week.

When assessed according to patterns of ACR50 response with guselkumab over time, statistically significant mean changes from baseline through Week 100, some as early as Week 4, were observed in levels of CRP, IL-6, SAA, C1M, C4M, and C6M in participants who achieved an ACR50 response at both Weeks 24 and 100 (R/R), and in those who improved from ACR50 nonresponder at Week 24 to ACR50 responder at Week 100 (NR/R; Figure 5). Although significant changes in biomarker levels were observed from baseline at Week 24 among participants of both ACR50 response cohorts, these changes were numerically lower for collagen biomarkers among ACR50 NR/R than R/R participants at both timepoints. In the ACR50 R/R cohort, C3M levels also significantly decreased from baseline to Week 100, and in the ACR50 NR/R cohort, C1M levels significantly decreased from Week 24 to Week 100.

Relative to ACR50 nonresponders at both Weeks 24 and 100 (NR/NR) in both biomarker cohorts, mean decreases from baseline at Week 100 for all biomarkers assessed were numerically greater among guselkumab-randomized ACR50 R/R and ACR50 NR/R participants (Figure 5). The reductions in CRP, SAA, IL-6, and C1M concentrations seen in ACR50 NR/R participants with guselkumab were statistically significantly greater than those seen in the ACR50 NR/NR cohort.

## Discussion

Serum biomarkers may have utility as objective measures of inflammation and disease activity and as predictors of treatment response in patients with PsA.^32–35^ Previous analyses from the phase III DISCOVER-2 study have demonstrated clinically meaningful and durable improvements in overall PsA disease activity and across PsA domains, such as achieving almost clear/clear skin, resolution of dactylitis and enthesitis, and minimal disease activity through 2 years of guselkumab treatment.^16,18,19,36^ Results of the current post hoc biomarker analyses using data from DISCOVER-2 further our understanding of the pharmacodynamic effects of guselkumab in biologic-naïve patients with active PsA and their relationships with clinical efficacy outcomes.

Consistent with the overall DISCOVER-2 population of biologic-naïve patients with active PsA, the evaluated biomarker cohorts were characterized by moderate-to-high levels of disease activity. Response rates for achieving ACR50 at Week 24 in the inflammatory (58%) and collagen (54%) biomarker cohorts were similar to those previously reported using nonresponder imputation and observed data from all patients randomized to the guselkumab Q4W (56% and 62%, respectively) and guselkumab Q8W (55% and 61%, respectively) groups in DISCOVER-2.^19^ The current literature describes the pathogenesis of PsA and psoriasis as driven by the IL-23/Th17 pathway in the joints and skin.^6–8,10,11,13,37,38^ Herein, baseline levels of IL-23/Th17 effector cytokines and BD-2 correlated with baseline skin disease activity. Despite a lack of association between baseline levels of the IL-23 effector cytokines IL-17A, IL-17F, and IL-22 with joint disease activity in the current analyses, improvements in this domain were observed through 2 years and correlated with baseline levels of acute phase proteins.

When assessing the long-term pharmacodynamic effects of guselkumab within the DISCOVER-2 biomarker cohorts, significant reductions in serum concentrations of acute phase proteins, IL-23/Th17 effector cytokines, BD-2, and collagen degradation biomarkers were observed with both the Q4W and Q8W dosing regimens. Participants in the guselkumab groups had significant reductions in serum SAA, IL-17A, IL-17F, IL-22, and BD-2 levels by Week 4; in CRP, IL-6, and C1M by Week 24; and in C6M by Week 100. Importantly, early pharmacodynamic effects were durable through 2 years of guselkumab treatment. These longer-term findings extend those from previous post hoc biomarker analyses of DISCOVER-1 and-2 through Week 24, in which guselkumab decreased IL-17A, IL-17F, and IL-22 to levels comparable to those seen in healthy controls^20^ and significantly decreased levels of collagen biomarkers at Week 24 compared with placebo, with maintenance of reduced levels at Week 52.^21^

Further advancing our understanding of relationships between guselkumab pharmacodynamic effects and durable clinical efficacy, reductions in acute phase protein and collagen degradation biomarkers correlated with concurrent improvements in joint (DAPSA and cDAPSA) and overall PsA (PASDAS) disease activity through 2 years, but not skin disease activity in the current analyses. By contrast, reductions in serum IL-17F, IL-22, and BD-2 levels with guselkumab correlated with improvements in PASI scores across timepoints assessed, but not in joint disease activity. These findings align with the known central role of IL-17 in the development of psoriatic skin lesions^12^ and suggest ongoing modulation of IL-23/Th17 pathway cytokines as the key mechanism underlying the durable improvements in skin disease afforded by guselkumab treatment. Interestingly, correlations between changes in both biomarker levels and disease activity were observed despite the nonsignificant pharmacodynamic effects of guselkumab on TNFα levels and a lack of association between changes in TNFα levels and clinical improvement through 2 years. These results are consistent with those of a previous biomarker analysis from DISCOVER-1, DISCOVER-2, and the phase III COSMOS study of guselkumab in TNFi-experienced adults with PsA. Briefly, TNFα levels did not decrease significantly with guselkumab treatment through Week 24, and baseline levels did not correlate with achievement of Week 24 clinical responses, apart from the Investigator’s Global Assessment of psoriasis score of 0/1 (clear/minimal) response among patients with an inadequate response to prior TNFi (TNFi-IR).^39^

Results of correlation analyses also expand our understanding of biomarkers as predictors of clinical response to guselkumab. Previously, in the pooled DISCOVER-1 and DISCOVER-2 biomarker cohort, baseline levels of CRP, SAA, and IL-6 did not predict ACR20 response at Week 24.^20^ However, in the current analyses, levels of these acute phase proteins at Week 24 in guselkumab-randomized DISCOVER-2 participants were significantly lower among Week 100 ACR50 responders versus nonresponders. In addition, reductions in these and other acute phase proteins and collagen biomarkers were significantly greater in participants who ultimately achieved an ACR50 response by Week 100 (NR/R) than in those who did not (NR/NR). Furthermore, smaller decreases in collagen biomarkers observed from baseline at Week 24 among participants improving from Week 24 ACR50 nonresponder to Week 100 responder (NR/R) compared with participants achieving an ACR50 response at both timepoints (R/R) suggest a subgroup of patients with PsA may exhibit protracted reduction of collagen biomarkers with guselkumab therapy, which is associated with delayed achievement of ACR50. Reductions from baseline in serum IL-6 levels at Week 24 with guselkumab also correlated with later improvements from baseline in DAPSA and cDAPSA scores at Week 100. Specific to skin psoriasis, reductions in serum IL-17A, IL-17F, IL-22, and BD-2 levels at Week 24 of guselkumab treatment correlated with improvements in PASI score at Week 100. Reductions in these biomarkers and improvements in PASI score also correlated with one another at pooled and individual timepoints through Week 100. The time course of relationships between biomarkers assessed and composite peripheral joint and skin disease assessments suggests separate molecular mechanisms by which guselkumab affords durable improvements in these key PsA domains and highlights the potential role of lasting cytokine suppression in the incremental benefits associated with continued guselkumab treatment.

Along with clinical outcomes, earlier identification of effective treatment options for patients may also improve long-term radiographic outcomes.^40^ Given its impact on physical function and health-related quality of life, preventing the onset or progression of structural damage is a key concern for patients with PsA.^41^ As previously reported, DISCOVER-2 participants exhibited higher baseline levels of collagen biomarkers, including C1M, relative to healthy controls.^21^ As an identified potential biomarker of bone turnover in rheumatoid arthritis,^42^ C1M may also serve as a biomarker of structural damage in PsA to help identify patients at increased risk. The significant and durable reductions in C1M levels with guselkumab treatment reported herein are consistent with previous findings that guselkumab-treated participants in DISCOVER-2 had low rates of radiographic progression through 2 years, and rates of radiographic progression were significantly lower at Week 24 with guselkumab Q4W and numerically lower with guselkumab Q8W compared with placebo.^16,18,19^ In addition, guselkumab-randomized participants who achieved low levels of disease activity had less radiographic progression than nonresponders at 1 and 2 years.^43^ In the current analyses, the associations observed between serum C1M levels and changes in peripheral joint disease activity (DAPSA) through 2 years with guselkumab may relate to decreases in inflammation-driven bone turnover.^21^ The ongoing phase IIIb clinical trial APEX (clinicaltrials.gov identifier: NCT04882098) will further assess the efficacy and pharmacodynamic effects of guselkumab over 3 years in a population of PsA patients exhibiting an elevated risk of radiographic progression.^44^

The long-lasting, and in some cases enhanced, pharmacodynamic effects of guselkumab reported herein also align with findings from previously conducted whole blood transcriptome profiling of DISCOVER-1 and -2 participants. Guselkumab treatment was shown to suppress inflammation-associated gene pathways upregulated in patients with active PsA, most notably genes involved in extracellular matrix organization and collagen formation, by Week 24.^38^ Greater modulation of upregulated PsA-associated genes was seen in both joint (ACR20) responders and skin (PASI75) responders than in nonresponders at Week 24. Within this framework, the partial normalization of transcriptomic signatures of immune cell genes by Week 24 of guselkumab may amplify its direct pharmacodynamic effects over time, with continued suppression of cytokine production leading to deeper and more durable levels of clinical response.

The unique molecular attributes of guselkumab, which enable potent neutralization of IL-23 at its cellular source, may also contribute to the substantial and durable multi-domain effectiveness of guselkumab.^36^ As a fully human IgG1 monoclonal antibody with a native Fc region, guselkumab simultaneously binds to the Fcγ receptor I (CD64) of primary human inflammatory monocytes in vitro and captures the IL-23 they secrete.^45^ Numbers of CD64+ IL-23-producing myeloid cells are increased in inflamed tissues of patients with psoriatic disease.^46^ Together with the positive correlations previously observed between the frequency of peripheral CD64+ monocytes and joint disease activity,^47^ guselkumab’s ability to downregulate myeloid gene sets upregulated in PsA may underpin the continued suppression of cytokine production needed to achieve low levels of disease activity and impede disease progression.^38^

This post hoc biomarker study of an IL-23p19-subunit inhibitor through 2 years is the first of its kind conducted in PsA. Strengths of these analyses include the high patient retention rate of DISCOVER-2 through 1 (94%) and 2 (90%) years for those randomized to guselkumab.^18,19^ Results of the current analysis further validate the robust and long-term efficacy of guselkumab seen in the DISCOVER-2 study^16,18,19^ and suggest potential predictors of long-term clinical improvement with guselkumab treatment as measured by improvement from ACR50 nonresponse at Week 24 to response at Week 100. Observed rates of ACR50 response, a stringent measure of joint disease improvement, with guselkumab in the biomarker cohorts were consistent with those of the overall DISCOVER-2 population through 2 years, suggesting the biomarker cohorts are representative of the overall DISCOVER-2 population.^19^

Several aspects of these post hoc analyses may limit their generalizability. Serum biomarker levels may not directly correspond with tissue levels. While the biomarker cohorts were of reasonable sample size, and baseline characteristics were consistent with the overall guselkumab-randomized DISCOVER-2 population, the numbers of patients comprising further subgroups (e.g., ACR50 responders and nonresponders at Week 100) were limited. In addition, possible effects of concomitant methotrexate or corticosteroids were not assessed owing to the small sample sizes and changes to concomitant medications permitted per protocol after Week 52. However, it should be noted that in pooled analyses from DISCOVER-1 and DISCOVER-2, response rates for achieving stringent levels of response across PsA domains were similar regardless of concomitant methotrexate use,^48^ and no associations were observed between concomitant methotrexate use and baseline levels of inflammatory biomarkers (data on file). Furthermore, although biomarker patterns and relationships with clinical improvements reported in the guselkumab Q4W and Q8W treatment groups were widely consistent with those of the combined guselkumab group, general conclusions cannot be drawn due to the limited sample sizes of the individual groups. As DISCOVER-2 enrolled only biologic-naïve participants who met the criteria for trial participation, including ⩾5 swollen and ⩾5 tender joints and serum CRP ⩾0.6 mg/dL, findings may not apply to the broader population of patients with PsA seen in clinical practice. Results from the ongoing APEX study (high risk of radiographic progression in PsA), together with those from the SOLSTICE^49^ (TNFi-IR PsA; NCT04936308) and STAR^50^ (axial PsA; NCT04929210) studies, will extend our understanding of biomarkers and the pharmacodynamic response of guselkumab among these particular PsA populations with high unmet need.

## Conclusion

Novel findings in the current analyses of the phase III DISCOVER-2 study provide evidence that the early and sustained reductions in serum levels of acute phase proteins, IL-23/Th17 effector cytokines, BD-2, and collagen degradation markers through 2 years with guselkumab therapy, and their association with the clinical efficacy assessments evaluated, support the robust, continuous, and long-lasting improvements in joint, skin, and overall PsA disease activity previously reported in these biologic-naïve patients with active PsA.^16,18,19,36^

## Supplemental Material

sj-docx-1-tab-10.1177_1759720X241283536 – Supplemental material for Correlation of changes in inflammatory and collagen biomarkers with durable guselkumab efficacy through 2 years in participants with active psoriatic arthritis: results from a phase III randomized controlled trial Supplemental material, sj-docx-1-tab-10.1177_1759720X241283536 for Correlation of changes in inflammatory and collagen biomarkers with durable guselkumab efficacy through 2 years in participants with active psoriatic arthritis: results from a phase III randomized controlled trial by Stefan Siebert, Georg Schett, Siba P. Raychaudhuri, Monica Guma, Warner Chen, Sheng Gao, Soumya D. Chakravarty, Frederic Lavie and Proton Rahman in Therapeutic Advances in Musculoskeletal Disease

sj-docx-2-tab-10.1177_1759720X241283536 – Supplemental material for Correlation of changes in inflammatory and collagen biomarkers with durable guselkumab efficacy through 2 years in participants with active psoriatic arthritis: results from a phase III randomized controlled trial Supplemental material, sj-docx-2-tab-10.1177_1759720X241283536 for Correlation of changes in inflammatory and collagen biomarkers with durable guselkumab efficacy through 2 years in participants with active psoriatic arthritis: results from a phase III randomized controlled trial by Stefan Siebert, Georg Schett, Siba P. Raychaudhuri, Monica Guma, Warner Chen, Sheng Gao, Soumya D. Chakravarty, Frederic Lavie and Proton Rahman in Therapeutic Advances in Musculoskeletal Disease

sj-docx-3-tab-10.1177_1759720X241283536 – Supplemental material for Correlation of changes in inflammatory and collagen biomarkers with durable guselkumab efficacy through 2 years in participants with active psoriatic arthritis: results from a phase III randomized controlled trial Supplemental material, sj-docx-3-tab-10.1177_1759720X241283536 for Correlation of changes in inflammatory and collagen biomarkers with durable guselkumab efficacy through 2 years in participants with active psoriatic arthritis: results from a phase III randomized controlled trial by Stefan Siebert, Georg Schett, Siba P. Raychaudhuri, Monica Guma, Warner Chen, Sheng Gao, Soumya D. Chakravarty, Frederic Lavie and Proton Rahman in Therapeutic Advances in Musculoskeletal Disease

sj-docx-4-tab-10.1177_1759720X241283536 – Supplemental material for Correlation of changes in inflammatory and collagen biomarkers with durable guselkumab efficacy through 2 years in participants with active psoriatic arthritis: results from a phase III randomized controlled trial Supplemental material, sj-docx-4-tab-10.1177_1759720X241283536 for Correlation of changes in inflammatory and collagen biomarkers with durable guselkumab efficacy through 2 years in participants with active psoriatic arthritis: results from a phase III randomized controlled trial by Stefan Siebert, Georg Schett, Siba P. Raychaudhuri, Monica Guma, Warner Chen, Sheng Gao, Soumya D. Chakravarty, Frederic Lavie and Proton Rahman in Therapeutic Advances in Musculoskeletal Disease

## Declarations

**Ethics approval and consent to participate:** DISCOVER-2 was conducted in accordance with the Declaration of Helsinki and Good Clinical Practice guidelines, and all patients gave written informed consent. The governing ethical bodies for each of the participating sites approved the DISCOVER-2 trial protocol (Sterling institutional review board approval number (US sites): 5910C). All study participants provided written informed consent before any study-related procedures were performed.

**Consent for publication:** Not applicable.

**Availability of data and materials:** The data sharing policy of Janssen Pharmaceutical Companies of Johnson & Johnson is available at https://www.janssen.com/clinicaltrials/transparency. As noted on this site, requests for access to the study data can be submitted through Yale Open Data Access (YODA) Project site at http://yoda.yale.edu.
