# Supplementary material for: Correlation of changes in inflammatory and collagen biomarkers with durable guselkumab efficacy through 2 years in participants with active psoriatic arthritis: results from a phase III randomized controlled trial
Source: Ther Adv Musculoskelet Dis. 2024 Oct 27;16:1759720X241283536. doi: 10.1177/1759720X241283536 (PMC11528637; doi:10.1177/1759720X241283536)
Supplement: sj-docx-1-tab-10.1177_1759720X241283536 – Supplemental material for Correlation of changes in inflammatory and collagen biomarkers with durable guselkumab efficacy through 2 years in participants with active psoriatic arthritis: results from a phase III randomized controlled trial [file sj-docx-1-tab-10.1177_1759720X241283536.docx]

| **Supplemental Table 1. Baseline demographic and disease characteristics: guselkumab (Q4W+Q8W)-randomized participants in the overall, inflammatory biomarker, and collagen biomarker cohorts of DISCOVER-2** | | | | | | | |
| --- | --- | --- | --- | --- | --- | --- | --- |
|  | **Overall DISCOVER-2** | | **Inflammatory biomarker cohort** | | | **Collagen biomarker cohort** | |
| **Baseline characteristic** | **GUS Q4W** | **GUS Q8W** | **GUS Q4W** | **GUS Q8W** | **GUS Q4W** | | **GUS Q8W** |
| **Participants,** n | 245 | 248 | 50 | 50 | 83 | | 95 |
| **Age,** years | 45.9 (11.5) | 44.9 (11.9) | 46.7 (9.9) | 45.9 (13.3) | 45.2 (10.8) | | 44.9 (11.6) |
| **Male,** % | 58.0 | 52.0 | 54.0 | 54.0 | 61.4 | | 54.7 |
| **White,** % | 98.8 | 96.8 | 98.0 | 98.0 | 98.8 | | 94.7 |
| **BMI,** kg/m^2^ | 29.1 (5.9) | 28.7 (6.3) | 28.8 (5.6) | 28.4 (5.6) | 29.4 (5.9) | | 28.6 (5.9) |
| **CRP,** mg/dL  Median (IQR)  Mean (SD) | 1.2 (0.02-19.0)  1.8 (2.2) | 1.3 (0.0-18.8)  2.0 (2.4) | 1.6 (0.0-10.3)  2.2 (2.3) | 1.4 (0.1-12.0)  2.0 (2.2) | 1.5 (0.1-19.0)  2.2 (2.6) | | 1.4 (0.0-10.3)  2.0 (2.0) |
| **SJC** [0-66] | 12.9 (7.8) | 11.7 (6.8) | 13.0 (8.4) | 12.9 (7.4) | 14.5 (9.0) | | 12.0 (7.7) |
| **TJC** [0-68] | 22.4 (13.5) | 19.8 (11.9) | 21.6 (13.2) | 22.3 (12.7) | 24.5 (14.4) | | 20.1 (12.3) |
| **DSS** [1-60]  **LEI** [1-6] | 8.6 (9.6)^a^  3.0 (1.7)^b^ | 8.0 (9.6)^a^  2.6 (1.5)^b^ | 8.7 (9.4)  2.9 (1.7) | 10.0 (11.0)  3.0 (1.7) | 10.0 (11.6)  3.0 (1.6) | | 8.4 (11.6)  2.4 (1.4) |
| **PhGA** (VAS) [0-10] | 6.6 (1.5) | 6.6 (1.6) | 6.8 (1.4) | 6.6 (1.6) | 6.8 (1.6) | | 6.8 (1.4) |
| **PtGA Arthritis** (VAS) [0-10] | 6.4 (1.9) | 6.5 (1.9) | 6.3 (2.2) | 6.8 (1.8) | 6.3 (1.9) | | 6.6 (1.7) |
| **PtGA Arthritis+PsO** (VAS) [0-100] | 67.1 (19.6) | 67.5 (20.5) | 66.7 (20.5) | 71.3 (18.0) | 67.0 (19.9) | | 67.9 (18.6) |
| **Patient Pain** (VAS) [0-10]  **HAQ-DI** [0-3] | 6.2 (2.0)  1.2 (0.6) | 6.3 (2.0)  1.3 (0.6) | 6.3 (2.2)  1.3 (0.6) | 6.5 (1.8)  1.3 (0.6) | 6.2 (1.9)  1.3 (0.6) | | 6.5 (1.7)  1.4 (0.6) |
| **DAPSA score** | 49.7 (21.1) | 46.3 (19.4) | 49.5 (22.2) | 50.5 (20.2) | 53.7 (23.1) | | 47.3 (20.3) |
| **cDAPSA score** | 47.9 (20.9) | 44.3 (18.8) | 47.3 (21.8) | 48.5 (19.8) | 51.5 (22.8) | | 45.2 (19.4) |
| **PASI score** [0-72] | 10.8 (11.7) | 9.7 (11.7) | 10.6 (13.0) | 9.8 (10.8) | 11.4 (11.5) | | 8.9 (10.8) |
| **PASDAS** [0-10] | 6.6 (1.1) | 6.6 (1.1) | 6.6 (1.1) | 6.7 (1.0) | 6.8 (1.1) | | 6.6 (1.0) |
| **Concomitant medications,** %  csDMARD  Methotrexate  Corticosteroid | 69.4  59.6  18.8 | 68.5  56.9  20.2 | 74.0  64.0  20.0 | 60.0  50.0  24.0 | 65.1  59.0  13.3 | | 73.7  61.1  23.2 |
| Data are mean (SD) unless otherwise indicated.  ^a^Among participants with dactylitis at baseline (overall DISCOVER-2: GUS Q4W N=121, GUS Q8W N=111; inflammatory biomarker cohort: GUS Q4W N=23, GUS Q8W N=21; collagen biomarker cohort: GUS Q4W N=44, GUS Q8W N=45.  ^b^Among participants with enthesitis at baseline (overall DISCOVER-2: GUS Q4W N=166, GUS Q8W N=157; inflammatory biomarker cohort: GUS Q4W N=34, GUS Q8W N=32; collagen biomarker cohort: GUS Q4W N=62, GUS Q8W N=61).  *BMI, body mass index; cDAPSA, clinical DAPSA; CRP, C-reactive protein; csDMARD, conventional synthetic disease-modifying antirheumatic drug; DAPSA, Disease Activity in PSoriatic Arthritis; DSS, dactylitis severity score; GUS, guselkumab; HAQ-DI, Health Assessment Questionnaire-Disability Index; IQR, interquartile range; LEI, Leeds enthesitis index; PASDAS, Psoriatic ArthritiS Disease Activity Score; PASI, Psoriasis Area and Severity Index; PhGA, Physician Global Assessment; PsO, psoriasis; PtGA, Patient Global Assessment; Q4W, every 4 weeks; Q8W, every 8 weeks; SD, standard deviation; SJC, swollen joint count; TJC, tender joint count; VAS, visual analog scale.* | | | | | | | |
