# Supplementary material for: Correlation of changes in inflammatory and collagen biomarkers with durable guselkumab efficacy through 2 years in participants with active psoriatic arthritis: results from a phase III randomized controlled trial
Source: Ther Adv Musculoskelet Dis. 2024 Oct 27;16:1759720X241283536. doi: 10.1177/1759720X241283536 (PMC11528637; doi:10.1177/1759720X241283536)
Supplement: sj-docx-2-tab-10.1177_1759720X241283536 – Supplemental material for Correlation of changes in inflammatory and collagen biomarkers with durable guselkumab efficacy through 2 years in participants with active psoriatic arthritis: results from a phase III randomized controlled trial [file sj-docx-2-tab-10.1177_1759720X241283536.docx]

| **Supplemental Table 2. Correlations between changes from baseline in inflammatory and collagen biomarker levels and changes in disease activity measures pooled across 3 timepoints (Week 24, Week 52, and Week 100): guselkumab Q4W- and guselkumab Q8W-randomized participants from the inflammatory and collagen biomarker cohorts of DISCOVER-2*** | | | | | | | | | |
| --- | --- | --- | --- | --- | --- | --- | --- | --- | --- |
|  |  | **DAPSA** | | **cDAPSA** | | **PASI** | | **PASDAS** | |
| **Time** | **Biomarker** | **GUS Q4W** | **GUS Q8W** | **GUS Q4W** | **GUS Q8W** | **GUS Q4W** | **GUS Q8W** | **GUS Q4W** | **GUS Q8W** |
| **Week 24** | **CRP** | 0.16 | **0.37** | 0.08 | **0.35** | -0.02 | -0.01 | 0.08 | 0.28 |
|  | **IL-6** | 0.22 | 0.28 | 0.16 | 0.27 | 0.07 | -0.12 | 0.19 | 0.19 |
|  | **SAA** | 0.14 | 0.24 | 0.06 | 0.21 | 0.01 | 0.06 | 0.22 | 0.19 |
|  | **TNFα** | -0.01 | 0.14 | -0.01 | 0.12 | **0.29** | -0.03 | 0.21 | 0.04 |
|  | **IL-17A** | 0.01 | 0.06 | -0.01 | 0.06 | **0.40** | **0.48** | **0.38** | 0.18 |
|  | **IL-17F** | -0.01 | 0.09 | -0.02 | 0.10 | **0.33** | **0.48** | 0.21 | 0.15 |
|  | **IL-22** | 0.21 | 0.08 | 0.20 | 0.11 | **0.41** | **0.39** | 0.23 | 0.12 |
|  | **BD-2** | 0.09 | 0.16 | 0.08 | 0.21 | **0.58** | **0.62** | 0.27 | **0.31** |
|  | **C1M** | 0.17 | **0.32** | 0.09 | **0.26** | -0.05 | 0.12 | 0.12 | 0.24 |
|  | **C3M** | 0.17 | **0.26** | 0.09 | 0.20 | -0.04 | 0.20 | 0.19 | **0.27** |
|  | **C4M** | 0.15 | **0.29** | 0.06 | 0.23 | 0.01 | 0.18 | 0.12 | **0.29** |
|  | **C6M** | **0.30** | **0.28** | 0.21 | 0.22 | -0.01 | 0.14 | 0.16 | **0.34** |
| **Week 52** | **CRP** | 0.32 | **0.37** | 0.24 | 0.29 | 0.03 | 0.12 | 0.26 | 0.15 |
|  | **IL-6** | **0.42** | 0.18 | **0.37** | 0.13 | 0.06 | -0.11 | 0.19 | 0.06 |
|  | **SAA** | 0.12 | 0.26 | 0.07 | 0.17 | 0.26 | 0.23 | **0.36** | 0.01 |
|  | **TNFα** | -0.13 | 0.29 | -0.17 | 0.20 | -0.29 | 0.18 | -0.16 | 0.17 |
|  | **IL-17A** | -0.11 | 0.13 | -0.13 | 0.06 | 0.27 | **0.57** | 0.09 | 0.07 |
|  | **IL-17F** | -0.11 | -0.11 | -0.13 | -0.13 | 0.34 | **0.52** | -0.07 | 0.03 |
|  | **IL-22** | -0.24 | -0.03 | -0.27 | -0.05 | 0.18 | **0.36** | -0.14 | -0.13 |
|  | **BD-2** | -0.04 | 0.14 | -0.05 | 0.14 | **0.40** | **0.66** | 0.35 | 0.09 |
|  | **C1M** | **0.27** | **0.28** | 0.19 | 0.21 | -0.06 | 0.17 | **0.28** | **0.31** |
|  | **C3M** | 0.21 | 0.19 | 0.13 | 0.13 | -0.02 | 0.04 | 0.25 | 0.18 |
|  | **C4M** | 0.21 | **0.31** | 0.12 | 0.24 | -0.06 | 0.13 | **0.34** | **0.28** |
|  | **C6M** | 0.20 | 0.20 | 0.13 | 0.14 | 0.06 | 0.09 | 0.24 | 0.23 |
| **Week 100** | **CRP** | 0.24 | **0.41** | 0.15 | **0.38** | 0.26 | 0.10 | **0.35** | **0.31** |
|  | **IL-6** | 0.25 | 0.19 | 0.19 | 0.16 | 0.15 | -0.21 | **0.31** | 0.11 |
|  | **SAA** | 0.26 | **0.37** | 0.18 | **0.35** | 0.25 | 0.15 | **0.39** | **0.31** |
|  | **TNFα** | 0.24 | 0.08 | 0.22 | 0.04 | 0.20 | 0.21 | **0.43** | 0.05 |
|  | **IL-17A** | 0.05 | 0.14 | 0.03 | 0.17 | **0.52** | **0.50** | 0.23 | 0.18 |
|  | **IL-17F** | -0.01 | -0.01 | 0.01 | -0.01 | **0.45** | **0.37** | 0.06 | 0.19 |
|  | **IL-22** | 0.11 | 0.01 | 0.10 | 0.01 | **0.34** | **0.31** | 0.24 | 0.04 |
|  | **BD-2** | 0.06 | -0.02 | 0.07 | 0.00 | **0.63** | **0.58** | 0.21 | 0.08 |
|  | **C1M** | 0.21 | 0.24 | 0.13 | 0.21 | 0.06 | 0.09 | **0.37** | 0.21 |
|  | **C3M** | 0.19 | 0.24 | 0.11 | 0.21 | 0.12 | 0.09 | **0.40** | 0.22 |
|  | **C4M** | 0.07 | 0.24 | -0.03 | 0.20 | 0.11 | 0.08 | **0.26** | 0.21 |
|  | **C6M** | 0.18 | **0.36** | 0.11 | **0.32** | -0.02 | 0.05 | 0.20 | **0.36** |
| **Pooled Week 24,**  **Week 52, &**  **Week 100** | **CRP** | 0.23 | **0.36** | 0.16 | **0.32** | 0.10 | 0.07 | **0.25** | 0.24 |
|  | **IL-6** | **0.30** | 0.20 | **0.25** | 0.17 | 0.08 | -0.16 | 0.24 | 0.10 |
|  | **SAA** | 0.16 | 0.23 | 0.09 | 0.18 | 0.15 | 0.13 | **0.28** | 0.14 |
|  | **TNFα** | 0.12 | 0.19 | 0.10 | 0.15 | 0.15 | 0.12 | **0.30** | 0.10 |
|  | **IL-17A** | 0.01 | 0.13 | -0.02 | 0.13 | **0.41** | **0.52** | **0.25** | 0.16 |
|  | **IL-17F** | -0.03 | 0.03 | -0.04 | 0.03 | **0.37** | **0.45** | 0.09 | 0.13 |
|  | **IL-22** | 0.16 | 0.18 | 0.14 | 0.19 | **0.34** | **0.33** | **0.27** | 0.19 |
|  | **BD-2** | 0.03 | 0.10 | 0.03 | 0.12 | **0.56** | **0.61** | 0.21 | 0.16 |
|  | **C1M** | 0.22 | **0.31** | 0.15 | **0.26** | -0.01 | 0.12 | **0.27** | **0.28** |
|  | **C3M** | 0.21 | **0.27** | 0.14 | 0.22 | 0.02 | 0.11 | **0.28** | **0.25** |
|  | **C4M** | 0.16 | **0.29** | 0.08 | 0.23 | 0.02 | 0.13 | 0.24 | **0.27** |
|  | **C6M** | **0.27** | **0.33** | 0.19 | **0.27** | 0.00 | 0.09 | 0.22 | **0.36** |
| *Among participants with available biomarker data (inflammatory biomarker cohort: GUS Q4W, N=50, GUS Q8W, N=50; collagen biomarker cohort: GUS Q4W N=83, GUS Q8W N=95).  Bolded rho (*r*) values represent statistically significant correlation between cytokine levels and clinical activity (*r*>0.25 and *p*<0.05).  *BD-2, β-defensin 2; C1M, MMP-degradation type 1 collagen; C3M, MMP-degradation type III collagen; C4M, MMP-degradation type IV collagen; C6M, MMP-degradation type VI collagen; cDAPSA, clinical DAPSA; CRP, C-reactive protein; DAPSA, Disease Activity in Psoriatic Arthritis; GUS, guselkumab; IL, interleukin; MMP, matrix metalloproteinase; PASDAS, Psoriatic Arthritis Disease Activity Score; PASI, Psoriasis Area and Severity Index; Q4W, every 4 weeks; Q8W, every 8 weeks; SAA, serum amyloid A; TNFα, tumor necrosis factor α.* | | | | | | | | | |
