# Supplementary material for: Correlation of changes in inflammatory and collagen biomarkers with durable guselkumab efficacy through 2 years in participants with active psoriatic arthritis: results from a phase III randomized controlled trial
Source: Ther Adv Musculoskelet Dis. 2024 Oct 27;16:1759720X241283536. doi: 10.1177/1759720X241283536 (PMC11528637; doi:10.1177/1759720X241283536)
Supplement: sj-docx-3-tab-10.1177_1759720X241283536 – Supplemental material for Correlation of changes in inflammatory and collagen biomarkers with durable guselkumab efficacy through 2 years in participants with active psoriatic arthritis: results from a phase III randomized controlled trial [file sj-docx-3-tab-10.1177_1759720X241283536.docx]

| **Supplemental Table 3. Correlations between changes in inflammatory and collagen biomarker levels and changes in disease activity through 2 years: guselkumab (Q4W+Q8W)-randomized participants from the inflammatory and collagen biomarker cohorts of DISCOVER-2*** | | | | | | | | | | | | |
| --- | --- | --- | --- | --- | --- | --- | --- | --- | --- | --- | --- | --- |
| **Time** | **Biomarker** | **CRP (mg/dL)** | **SJC**  **[0-66]** | **TJC**  **[0-68]** | **DSS [0-20]** | **LEI**  **[0-6]** | **PhGA**  **(VAS, 0-100)** | **PtGA Arthritis (VAS, 0-10)** | **PtGA Arthritis+PsO (VAS, 0-100)** | **HAQ-DI [0-3]** | **Patient Pain (VAS, 0-10)** | **SF-36 PCS** |
| **Week 24** | **CRP** | – | 0.12 | 0.17 | 0.09 | 0.19 | 0.06 | 0.09 | 0.10 | 0.17 | 0.11 | -0.20 |
|  | **IL-6** | **0.48** | 0.13 | 0.15 | 0.13 | 0.23 | -0.01 | 0.12 | 0.12 | 0.30 | 0.17 | **-0.26** |
|  | **SAA** | **0.56** | 0.01 | 0.10 | 0.07 | 0.24 | 0.08 | 0.11 | 0.13 | 0.17 | 0.05 | -0.05 |
|  | **TNFα** | 0.16 | 0.16 | 0.03 | 0.15 | 0.15 | 0.07 | -0.12 | -0.01 | -0.07 | -0.07 | 0.02 |
|  | **IL-17A** | -0.01 | 0.16 | -0.06 | 0.19 | 0.00 | 0.21 | 0.07 | 0.22 | 0.11 | 0.18 | -0.15 |
|  | **IL-17F** | 0.04 | 0.05 | -0.03 | 0.13 | 0.02 | 0.06 | 0.03 | 0.22 | -0.01 | 0.09 | -0.02 |
|  | **IL-22** | 0.06 | 0.13 | 0.09 | -0.03 | -0.03 | 0.17 | -0.03 | 0.12 | 0.08 | 0.03 | 0.04 |
|  | **BD-2** | -0.05 | 0.15 | 0.08 | 0.17 | 0.03 | 0.18 | 0.04 | **0.26** | 0.13 | 0.08 | -0.08 |
|  | **C1M** | **0.68** | 0.17 | 0.11 | -0.06 | 0.08 | 0.15 | 0.12 | 0.14 | 0.03 | 0.14 | -0.15 |
|  | **C3M** | **0.62** | 0.16 | 0.06 | -0.03 | 0.07 | 0.09 | 0.12 | 0.16 | 0.02 | 0.16 | -0.16 |
|  | **C4M** | **0.64** | 0.13 | 0.10 | -0.04 | 0.07 | 0.11 | 0.11 | 0.17 | 0.06 | 0.14 | -0.18 |
|  | **C6M** | **0.65** | 0.17 | 0.13 | -0.06 | 0.07 | 0.16 | 0.22 | 0.24 | 0.21 | **0.27** | **-0.26** |
| **Week 52** | **CRP** | – | 0.24 | **0.25** | 0.13 | 0.20 | -0.04 | 0.07 | 0.11 | 0.19 | 0.10 | -0.19 |
|  | **IL-6** | **0.52** | 0.11 | 0.24 | 0.08 | -0.02 | -0.04 | 0.15 | 0.14 | 0.24 | 0.24 | -0.20 |
|  | **SAA** | **0.67** | 0.10 | 0.14 | 0.04 | 0.23 | 0.08 | 0.06 | 0.15 | 0.09 | 0.06 | -0.06 |
|  | **TNFα** | **0.34** | 0.17 | 0.05 | -0.01 | -0.01 | 0.02 | -0.15 | -0.11 | 0.05 | -0.07 | 0.10 |
|  | **IL-17A** | **0.30** | 0.02 | -0.01 | 0.06 | 0.16 | 0.04 | -0.07 | 0.15 | 0.05 | -0.09 | 0.07 |
|  | **IL-17F** | 0.18 | -0.05 | -0.11 | -0.10 | 0.06 | 0.02 | -0.04 | 0.14 | -0.05 | -0.07 | 0.16 |
|  | **IL-22** | 0.15 | -0.02 | 0.02 | -0.19 | 0.15 | -0.03 | -0.22 | -0.09 | -0.04 | -0.21 | 0.02 |
|  | **BD-2** | 0.21 | 0.04 | 0.07 | 0.15 | -0.02 | 0.10 | 0.05 | **0.27** | -0.04 | 0.03 | -0.03 |
|  | **C1M** | **0.72** | 0.13 | 0.08 | 0.09 | 0.10 | 0.14 | 0.15 | 0.21 | 0.04 | **0.28** | **-0.26** |
|  | **C3M** | **0.65** | 0.10 | 0.03 | -0.01 | 0.15 | 0.12 | 0.14 | 0.17 | -0.04 | 0.22 | -0.20 |
|  | **C4M** | **0.70** | 0.14 | 0.08 | 0.11 | 0.13 | 0.19 | 0.20 | **0.26** | 0.04 | **0.30** | -0.22 |
|  | **C6M** | **0.66** | 0.06 | 0.04 | 0.08 | 0.16 | 0.17 | 0.19 | 0.20 | 0.03 | **0.28** | **-0.25** |
| **Week 100** | **CRP** | – | 0.10 | **0.25** | 0.19 | 0.14 | 0.17 | **0.28** | **0.28** | **0.27** | 0.24 | **-0.26** |
|  | **IL-6** | **0.49** | 0.02 | 0.14 | 0.21 | -0.08 | 0.08 | 0.18 | 0.19 | **0.32** | **0.25** | **-0.30** |
|  | **SAA** | **0.67** | 0.03 | **0.25** | 0.10 | 0.12 | **0.27** | 0.06 | **0.36** | 0.24 | **0.31** | **-0.26** |
|  | **TNFα** | **0.32** | 0.10 | 0.14 | 0.18 | 0.20 | 0.21 | 0.02 | 0.20 | 0.17 | 0.14 | -0.08 |
|  | **IL-17A** | 0.09 | 0.18 | 0.09 | 0.24 | 0.12 | 0.23 | 0.09 | 0.24 | 0.17 | 0.09 | 0.01 |
|  | **IL-17F** | 0.07 | 0.15 | 0.00 | 0.17 | -0.02 | 0.06 | 0.02 | 0.15 | 0.05 | -0.03 | 0.04 |
|  | **IL-22** | 0.07 | 0.21 | 0.00 | 0.08 | 0.10 | **0.26** | 0.01 | 0.15 | 0.10 | 0.05 | 0.09 |
|  | **BD-2** | 0.08 | 0.14 | -0.01 | 0.17 | -0.02 | 0.10 | 0.04 | 0.21 | -0.03 | 0.03 | 0.15 |
|  | **C1M** | **0.66** | 0.13 | 0.10 | -0.01 | 0.06 | **0.28** | 0.20 | 0.16 | 0.23 | 0.21 | -0.22 |
|  | **C3M** | **0.54** | 0.23 | 0.08 | 0.03 | 0.11 | 0.16 | 0.17 | 0.14 | 0.19 | 0.20 | -0.18 |
|  | **C4M** | **0.66** | 0.12 | 0.02 | -0.05 | 0.04 | 0.18 | 0.19 | 0.16 | 0.19 | 0.21 | -0.18 |
|  | **C6M** | **0.61** | 0.21 | 0.08 | 0.02 | 0.09 | 0.17 | 0.23 | 0.15 | 0.25 | **0.27** | **-0.26** |
| **Pooled Week 24,**  **Week 52, &**  **Week 100** | **CRP** | – | 0.14 | 0.22 | 0.14 | 0.18 | 0.08 | 0.16 | 0.19 | 0.21 | 0.16 | -0.23 |
|  | **IL-6** | **0.50** | 0.08 | 0.19 | 0.16 | 0.04 | 0.01 | 0.13 | 0.14 | **0.28** | 0.19 | -**0.25** |
|  | **SAA** | **0.63** | 0.01 | 0.14 | 0.07 | 0.19 | 0.11 | 0.17 | 0.21 | 0.16 | 0.13 | -0.12 |
|  | **TNFα** | **0.25** | 0.19 | 0.14 | 0.13 | 0.14 | 0.16 | -0.03 | 0.07 | 0.07 | 0.03 | -0.02 |
|  | **IL-17A** | 0.11 | 0.14 | 0.01 | 0.18 | 0.09 | 0.19 | 0.07 | 0.22 | 0.14 | 0.10 | -0.04 |
|  | **IL-17F** | 0.09 | 0.07 | -0.03 | 0.09 | 0.02 | 0.07 | 0.03 | 0.18 | 0.01 | 0.02 | 0.04 |
|  | **IL-22** | 0.07 | 0.22 | 0.14 | 0.02 | 0.11 | **0.26** | 0.05 | 0.17 | 0.11 | 0.07 | -0.01 |
|  | **BD-2** | 0.06 | 0.11 | 0.04 | 0.17 | 0.00 | 0.13 | 0.04 | 0.23 | 0.04 | 0.05 | 0.02 |
|  | **C1M** | **0.68** | 0.16 | 0.12 | 0.01 | 0.08 | 0.21 | 0.18 | 0.19 | 0.12 | 0.23 | -0.22 |
|  | **C3M** | **0.60** | 0.18 | 0.09 | 0.00 | 0.12 | 0.14 | 0.16 | 0.18 | 0.08 | 0.21 | -0.19 |
|  | **C4M** | **0.67** | 0.14 | 0.08 | 0.01 | 0.08 | 0.17 | 0.17 | 0.20 | 0.11 | 0.22 | -0.20 |
|  | **C6M** | **0.64** | 0.18 | 0.12 | 0.02 | 0.11 | 0.20 | **0.25** | 0.23 | 0.20 | **0.30** | **-0.28** |
| *Among participants with available biomarker data (inflammatory biomarker cohort, N=100; collagen biomarker cohort, N=178).  Statistics based on Spearman linear regression.  Bolded rho (*r*) values represent statistically significant correlations between cytokine levels and clinical activity (*r*>0.25 and *p*<0.05).  *BD-2, β-defensin 2; C1M, MMP-degradation type 1 collagen; C3M, MMP-degradation type III collagen; C4M, MMP-degradation type IV collagen; C6M, MMP-degradation type VI collagen; CRP, C-reactive protein; DSS, dactylitis severity score; HAQ-DI, Health Assessment Questionnaire – Disability Index; IL, interleukin; LEI, Leeds Enthesitis Index; MMP, matrix metalloproteinase; ; PhGA, Physician Global Assessment; PsO, psoriasis; PtGA, Patient Global Assessment; Q4W, every 4 weeks; Q8W, every 8 weeks; SAA, serum amyloid A; SF-36 PCS, 36-item Short-Form Health Survey Physical Component Summary; SJC, swollen joint count (66 joints); TJC, tender joint count (68 joints); TNFα, tumor necrosis factor α; VAS, visual analog scale.* | | | | | | | | | | | | |
