# Supplementary material for: Correlation of changes in inflammatory and collagen biomarkers with durable guselkumab efficacy through 2 years in participants with active psoriatic arthritis: results from a phase III randomized controlled trial
Source: Ther Adv Musculoskelet Dis. 2024 Oct 27;16:1759720X241283536. doi: 10.1177/1759720X241283536 (PMC11528637; doi:10.1177/1759720X241283536)
Supplement: sj-docx-4-tab-10.1177_1759720X241283536 – Supplemental material for Correlation of changes in inflammatory and collagen biomarkers with durable guselkumab efficacy through 2 years in participants with active psoriatic arthritis: results from a phase III randomized controlled trial [file sj-docx-4-tab-10.1177_1759720X241283536.docx]

**Supplemental Figure 1. Serum levels of inflammatory biomarkers from baseline through Week 100: guselkumab Q4W- and guselkumab Q8W-randomized participants from the inflammatory biomarker cohort of DISCOVER-2**. Statistics are based on a general linear model. *Indicates statistically significant change from baseline for guselkumab Q4W (*p*<0.05 and **|**fold difference**|** ≥1.4). ^†^Indicates statistically significant change from baseline for guselkumab Q8W (*p*<0.05 and **|**fold difference**|** ≥1.4).

*BD-2, β-defensin 2; CRP, C-reactive protein; GUS, guselkumab; IL, interleukin; Q4W, every 4 weeks; Q8W, every 8 weeks; SAA, serum amyloid A; SE, standard error; TNFα, tumor necrosis factor α.*

**
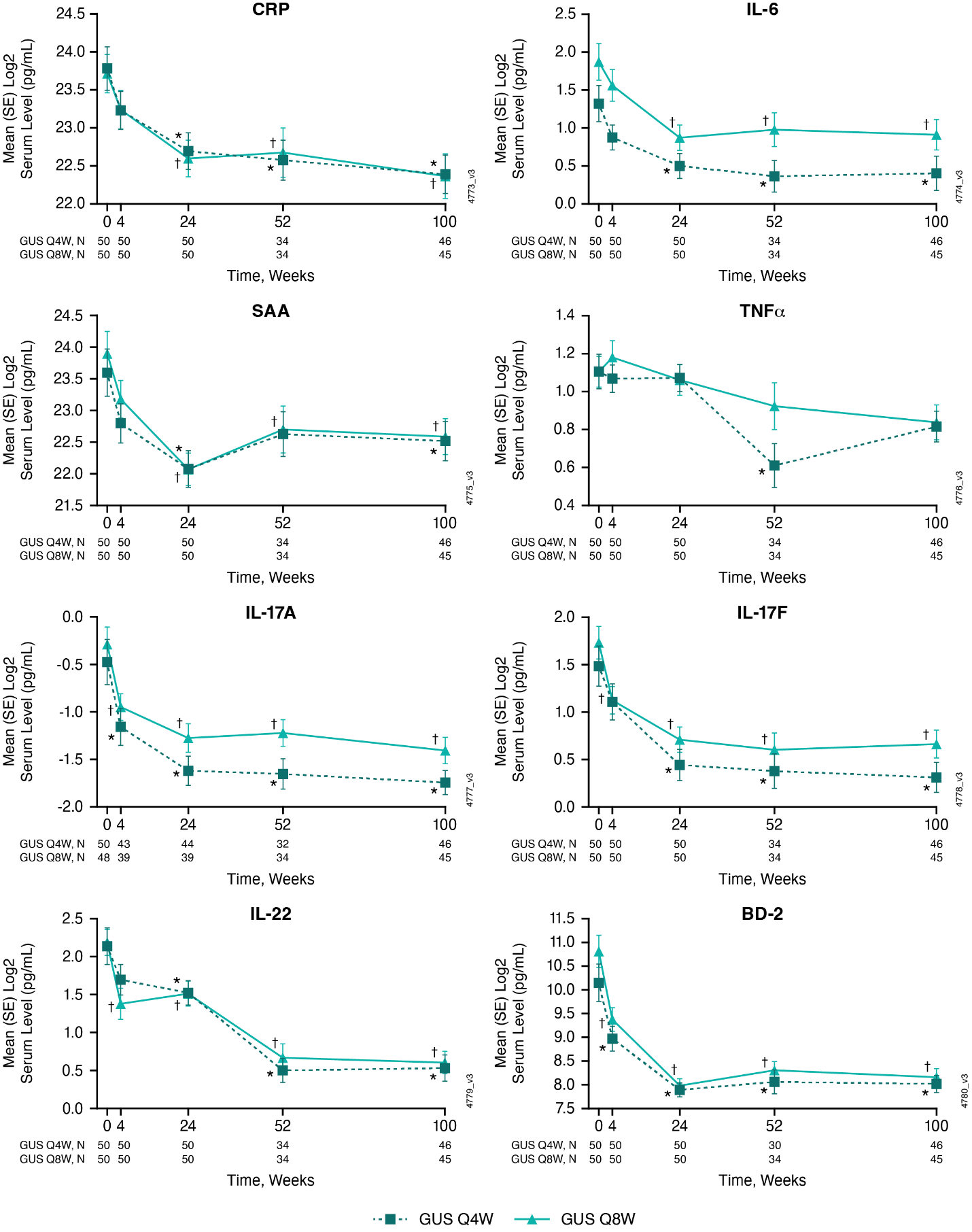
**

**Supplemental Figure 2. Serum levels of collagen biomarkers from baseline through Week 100: guselkumab Q4W- and guselkumab Q8W-randomized participants from the collagen biomarker cohort of DISCOVER-2**. Statistics are based on a general linear model. ^*^Indicates statistically significant change from baseline for combined guselkumab Q4W (*p*<0.05 and **|**fold difference**|** ≥1.25). ^†^Indicates statistically significant change from baseline for guselkumab Q8W (*p*<0.05 and **|**fold difference**|** ≥1.25).

*C1M, matrix metalloproteinase (MMP)-degradation type 1 collagen; C3M, MMP-degradation type III collagen; C4M, MMP-degradation type IV collagen; C6M, MMP-degradation type VI collagen; GUS, guselkumab; Q4W, every 4 weeks; Q8W, every 8 weeks; SE, standard error.*

**
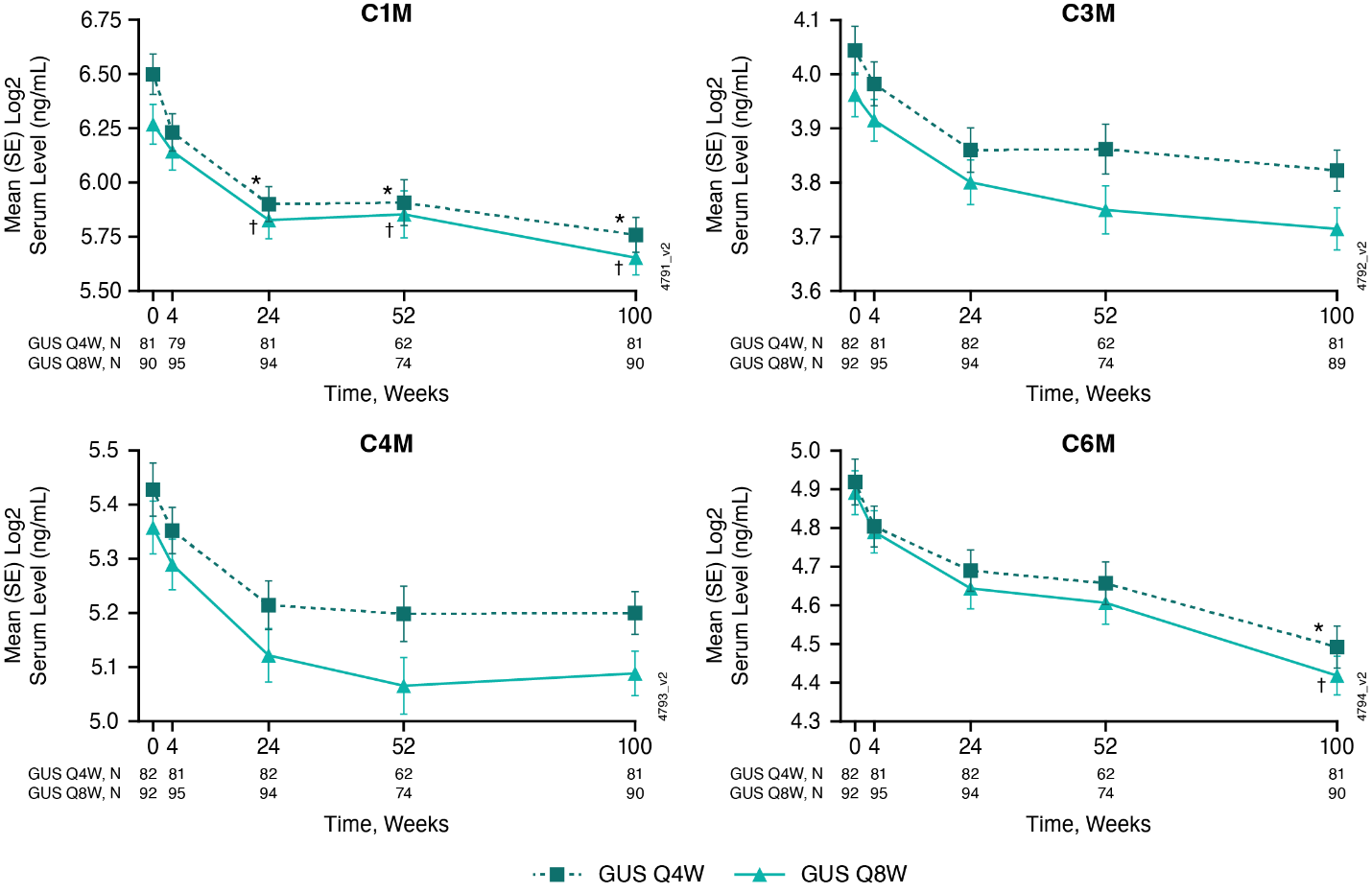
**
